# Toxicity, arsenic speciation and characteristics of hyphenated techniques used for arsenic determination in vegetables. A review

**DOI:** 10.1039/d3ra05770d

**Published:** 2023-10-23

**Authors:** Bashdar Abuzed Sadee, Yaseen Galali, Salih M. S. Zebari

**Affiliations:** a Department of Food Technology, College of Agricultural Engineering Sciences, Salahaddin University-Erbil KRG Iraq bashdar.sadee@su.edu.krd; b Department of Nutrition and Dietetics, Cihan University-Erbil Erbil Iraq; c Department of Animal Resource, College of Agricultural Engineering Sciences, Salahaddin University-Erbil KRG Iraq

## Abstract

Arsenic (As) speciation is an interesting topic because it is well recognized that the toxicity of this metalloid ultimately depends on its chemical form. More than 300 arsenicals exist naturally. However, As can be present in four oxidation states: As^−III^, As^0^, As^III^ and As^V^. Long-term exposure to As from different sources, such as anthropogenic processes, or water, fauna and flora contaminated with As, has put human health at risk for decades. There are many side-effects correlated with exposure to InAs species, such as skin problems, respiratory diseases, kidney problems, cardiovascular diseases and even cancer. There are different levels and types of As in foods, particularly in vegetables. Furthermore, different chemical methods and techniques have been developed. Therefore, this review focuses on the general properties of various approaches used to identify As species in vegetation samples published worldwide. This includes various approaches (different solvents and techniques) used to extract As species from the matrix. Then, versatile chromatographic and non-chromatographic systems to separate different forms of As are reviewed. Finally, the general properties of the most common instruments used to detect As species from samples of interest are listed.

## Abstract 2

Arsenic (As) speciation is an interesting topic because it is well recognized that the toxicity of this metalloid ultimately depends on its chemical form.

## Graphical abstract

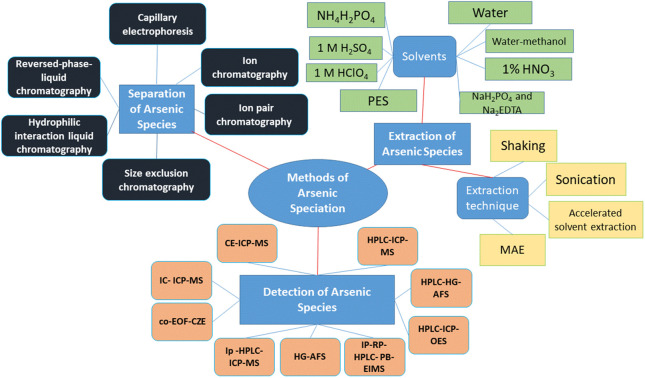

## 1 Introduction

Vegetables are recognized as crucial parts of the human diet, because they are a rich source of key elements like vitamins, minerals, dietary fiber, and antioxidants. Consuming vegetables on a daily basis, in both raw and cooked states, is good for human growth and development as well as for preventing various diseases,^1^ particularly when significant global challenges are food instability, malnutrition, and food-related diseases, including diabetes, high blood pressure, cancer, and obesity. These issues have led to an increase in the demand for healthy foods, particularly vegetables. It also encourages the production and consumption of vegetables, which account for 86% of global market share.^2^

The accumulation of heavy metals and metalloids in plants and vegetables has recently received increased attention. Due to the anthropogenic activities , vegetables may be more exposed to environmental contamination than other food systems. When heavy metals are accumulated and taken up by plants in edible and non-edible fractions at a particular level, both animals and people may experience health issues.^3^ Environmental trace elements are harmful to human health. Numerous studies have demonstrated that these factors can have an impact on the environment and the quality of food. It is commonly recognized that heavy metal contamination can pollute human food supplies, particularly vegetables.^4^ Plants may become contaminated by metals or microbial development due to a variety of causes, such as the environment, pollution, atmosphere, soil, harvesting, and handling. Determining the quantity of certain metallic elements is crucial, since consuming large amounts of these elements is harmful. The World Health Organization advises adopting appropriate methods and standard measurements to assure the quality of plants and their products.^5^

As pollution is a recognized human carcinogen that affects hundreds of millions of people worldwide. Inorganic As is a prominent cause of skin, lung, bladder, liver, prostate, and kidney cancer in humans.^6^ Because it is introduced to the environment both naturally and *via* human activity, As is a common substance.^7^ It exists in the pedosphere, hydrosphere, biosphere, atmosphere, and water. The biogeochemical behaviour of As is governed by physical–chemical processes, including oxidation–reduction, precipitation/solubilization, and adsorption/desorption in addition to biological mechanisms, including microbiological processes.^8^

Heavy metal exposure in vegetables can result from anthropogenic or natural processes. In contrast to anthropocentric metal concentration, naturally occurring metals are found in crusted materials, vapors, and particle matter from volcanoes and continental dust. The most significant and frequent sources of metals in vegetables come from anthropogenic activities, such as extensive long-term pesticide and fertilizer usage, as well as linear, point, and surface metal emissions from industrial activities.^9^ Additionally, the accumulation of As in environmental samples results from both manmade (using As-based insecticides and herbicides, primarily monomethyl arsenic acid (MMA) and dimethyl arsenic acid (DMA)) and natural (volcanic eruptions) sources. Arsenic trioxides [As_2_O_3_], which are used in cosmetics, fireworks, electronics, glass, Cu-based alloys, herbicides, fertilizers, pesticides, and seaweed fertilizers, are examples of As oxides used in industry, mining, agriculture, and other fields.^10^

**Fig. 1:**
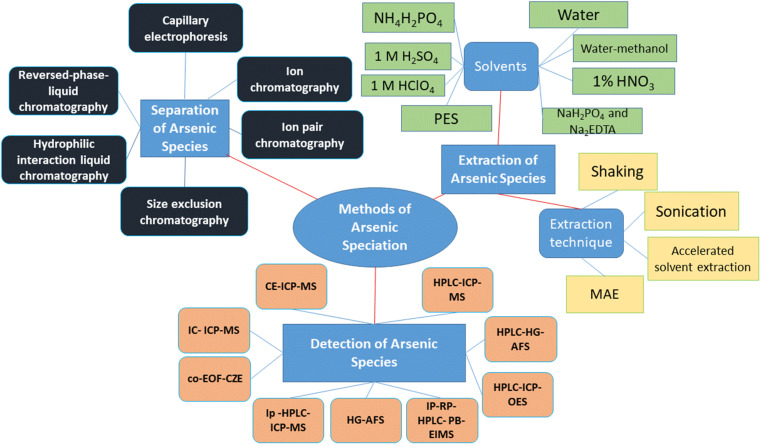
Different methods used to extract, separate and detect As species in vegetables.

Therefore, this review article summarizes the methods used to extract As species from vegetables, then separating them using chromatographic and non-chromatographic tools, followed by determining the level of As as well as speciating the types using hyphenated techniques (Fig. 1).

**Fig. 2:**
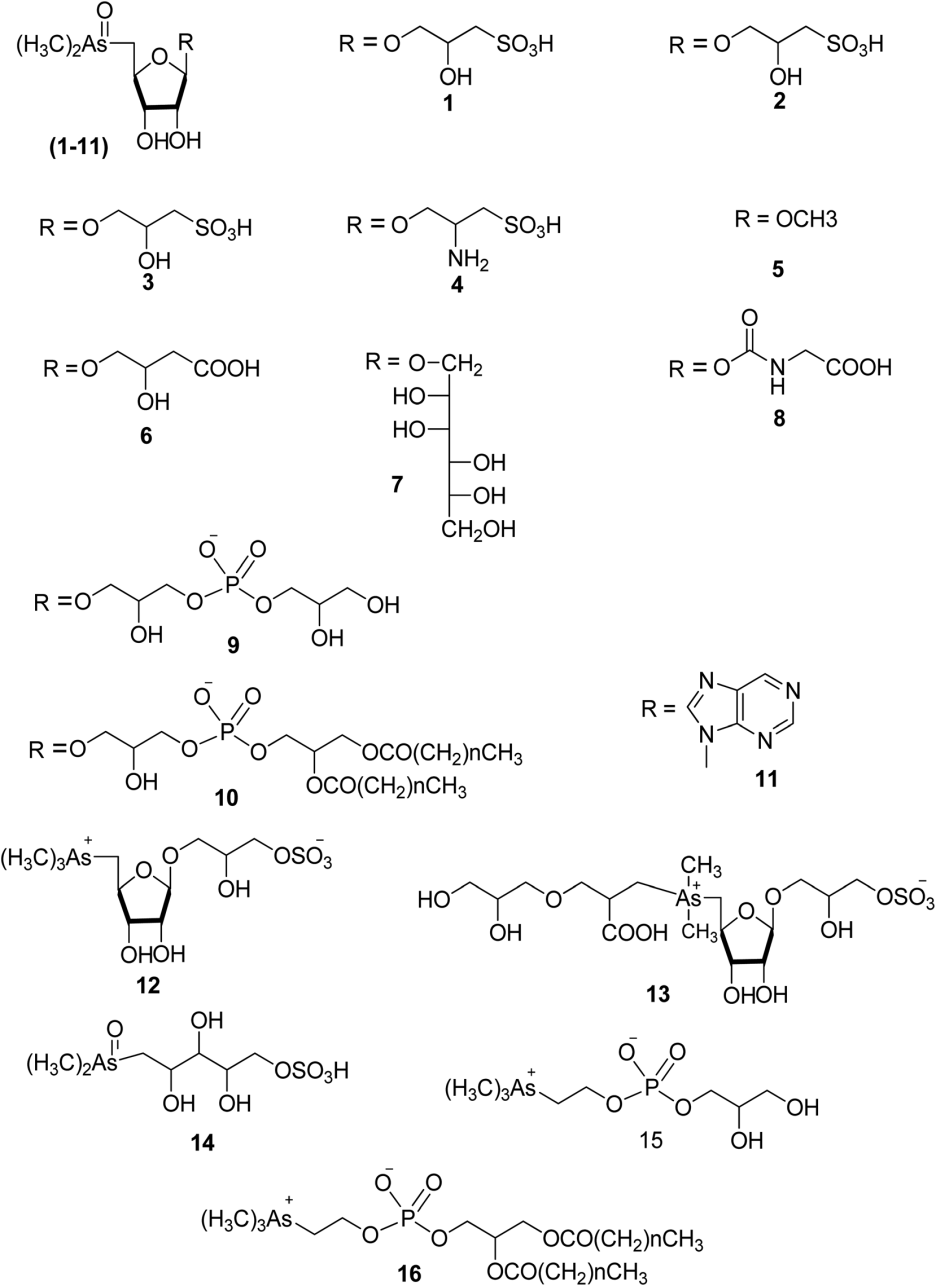
Examples of structures of arsenosugars and arsenolipids, 1–16 of Table 1.

## 2 Geochemistry of As

As is the 12^th^ most abundant metalloid in the human body and 20^th^ most abundant in nature. As and its derivatives are widely employed in a variety of industries, including agriculture, electronics, metallurgy, and chemical weapons, cattle, insecticides, and fertilizers, in addition to being used as a medicine.^11,12^ InAs compounds are produced when the element As, which is abundant in the earth's crust at 1.8 ppm by weight, mixes with oxygen, chlorine, and sulfur. The main source of As release and groundwater quality degradation in aquifer systems is rock-water interactions. As typically takes the three allotropic forms of black, yellow, and grey.^12^ As is an essential component of at least 568 minerals, which are found naturally in the minerals that comprise rocks.^10^ About 60% of these are arsenates, 20% are sulfides and sulfosalts, 10% are oxides, and the remainder are native elements, metal alloys, arsenites, and arsenides. The principal As-bearing minerals that include As as an anion (arsenide), dianion (diarsenide), or as a sulfarsenide anion or anion(s) are the most significant. These anions are bound to metals such Fe (löllingite, arsenopyrite), Co (cobaltite), and Ni (gersdorffite).^13^ The most important As-bearing minerals are orpiment (As_2_S_3_), realgar (AsS), mispickel (FeAsS), löllingite (FeAs_2_), niccolite (NiAs), cobaltite (CoAsS), tennantite (Cu_12_As_4_S_13_), and enargite (Cu_3_AsS_4_).^14^ Generally, in groundwater, high levels of naturally occurring As have been reported in aquifers—especially unconsolidated sediment aquifers throughout the world—and have been connected to several adverse health effects.^15^

## 3 As species

A significant part of the human diet is vegetables which can collect As in their edible and non-edible compartments by absorbing it from polluted agricultural soil and water.^16^ There are numerous inorganic and organic forms of As with different toxicity characteristics. As^0^ (metalloid arsenic, 0 oxidation state), As^III^ (trivalent state, *e.g.* arsenites), As^−III^ (trivalent state, arsine and arsenide, −3 oxidation state) and As^V^ (pentavalent state, *e.g.* arsenates) are the three most common valence states in which it can be found.^11,17^ A naturally occurring and widely dispersed metalloid, As can be found in soil, water, food, and the environment. As exposure from numerous human activities and sources, such as contaminated groundwater, has grown to be a major global concern. This is due to evidence that As has very hazardous potential to cause detrimental effects on human health. Human exposure to it has been connected to a wide range of diseases, and this poses a serious threat to people's health, economic security, and social standing, particularly in less developed nations.^17^ Even though As has a reputation for being hazardous, it is generally known that its toxicity relies greatly on the chemical state in which it exists and that the inorganic species As^III^ and As^V^ are considered to be more toxic than organoarsenic compounds (*e.g.* DMA and MMA).^18^Table 1 shows some of the most common As species.

**Table 1:** Some As compounds of environmental interest

Name	Chemical formula or structure
Arsenious acid	H_3_AsO_3_
Arsenic acid	H_3_AsO_4_
Methyl arsine	CH_3_AsH_2_
Dimethylarsine	(CH_3_)_2_AsH
Trimethylarsine	(CH_3_)_3_As
Monomethyl arsenic acid	CH_3_AsO(OH)_2_
Monomethylarsenous acid	CH_3_As(OH)_2_
Dimethyl arsenic acid	(CH_3_)_2_AsOH
Dimethylarsenous acid	(CH_3_)_2_AsOH
Trimethylarsinic oxide	TMAO
Tetramethylarsonium ion	TMA^+^
Arsenobetaine	(CH_3_)_3_As^+^CH_2_COO^−^
Arsenocholine	(CH_3_)_3_As^+^CH_2_CH_2_OH
Dimethylarsinoylribosides	See structures 1–11 in Fig. 2
Trialklylarsonioribosides	See structures 12, 13 in Fig. 2
Dimethylarsonoulribtol	See structure 14 in Fig. 2
Glecerophosphorarsnocholine	See structure 15 in Fig. 2
Glecerophosphorarsenocholine	See structure 16 in Fig. 2

## 4 Allowable limit of As in vegetation

Many international organizations have established recommended upper limits for the amount of As that should be present in foods. This is due to the heavy enrichment and biotransformation of As and its detrimental effects on human health. Any age or health condition can be negatively impacted by As, which is harmful to humans. Inorganic As (InAs) is the category of As that has the greatest risk of toxicity. As levels are monitored and controlled by the FDA in meals, nutritional supplements, and cosmetics. As cannot be completely prevented or removed from food, although its levels can be brought down. The EU has not yet defined a maximum limit for As in food products. However, in some states, the upper limits are set forth in national legislation. For instance, in the UK, 1 μg g^−1^ fresh weight of As is the statutory maximum for foods.^19^ The FDA is recommending a limit or “action level” for InAs in infant rice cereal of 100 μg kg^−1^. The standard set by the European Commission (EC) for rice used in the manufacturing of food for babies and young children is comparable to this.^20^ The Scientific Panel on Contaminants in the Food Chain (CONTAM Panel) of the European Food Safety Authority (EFSA) adopted an opinion on As in food on 12 October 2009. In this opinion, the CONTAM Panel stated that the Joint FAO/WHO Expert Committee on Food Additives' (JECFA) provisional tolerable weekly intake (PTWI) of 15 μg kg^−1^ body weight is no longer appropriate because data have shown that InAs causes cancer of the lung and bladder in addition to skin cancer and that a variety of adverse effects have been reported at exposures lower than those examined by the JECFA. For skin lesions, bladder, lung, and skin cancers, the CONTAM Panel determined a range of benchmark dose lower confidence limits (BMDL01) values between 0.3 and 8 μg kg^−1^ b.w. per day. The maximum values of InAs for non-parboiled milled rice (polished or white rice); parboiled rice and husked rice; rice waffles, rice wafers, rice crackers and rice cakes; and rice for the manufacturing of foods for infants and children were proposed by the EFSA to be 200, 250, 300, and 100 μg kg^−1^, respectively,^21^ China has established a legal cap of 150 μg kg^−1^ for InAs in rice and rice-based products.^22^ However, As exists at low concentrations in natural water, and in some locations, the levels of As in drinking water are over the maximum allowable concentration, which is 50 μg L^−1^, whereas the recommended threshold is 10 μg L^−1^, according to the Environmental Protection Agency (EPA).^23^

## 5 Toxicity of As

Elemental speciation has been a well-established subject of study in recent years. The mobility, biological availability, and toxicity of an element can be better understood by studying it in its chemical form.^24–26^ As is typically thought to be metabolized mostly through methylation to lessen its toxicity and this is considered to be a detoxification process. However, by 2020, it was discovered that the synthesis of methylated metabolites containing trivalent As was necessary for at least some of the harmful consequences linked to As exposure. These results were in line with changes in the dynamic behaviour of As brought on by methylation because the trivalent oxidation state of As is linked to increased effectiveness as a cytotoxin and clastogen.^27^

The InAs species (As^III^ and As^V^) are categorized as species that cause cancer.^28^ Whereas the organic As (OAs) species (MMA and DMA), despite being less toxic than InAs, are nevertheless categorized as species that provoke cancer,^29,30^ while AsC and AsB are categorized as non-toxic As species.^31^ However, intermediate metabolites, such as monomethylarsonous acid (MMA^III^) and dimethylarsinous acid (DMA^III^), are more toxic than As^III^, As^V^, DMA^V^ or MMA^V^.^32^

### 5.1 As and human health

Researchers have identified As as a significant danger factor for both food and water. It exists both naturally and as a result of human activity in the environment in organic and inorganic forms. Exposure to As may have negative health consequences on people as well as other living things. It can also have a variety of side effects, such as skin changes, respiratory issues, cardiovascular problems, digestive system issues, genotoxicity, and mutagenic and carcinogenic effects.^16^

**Fig. 3:**
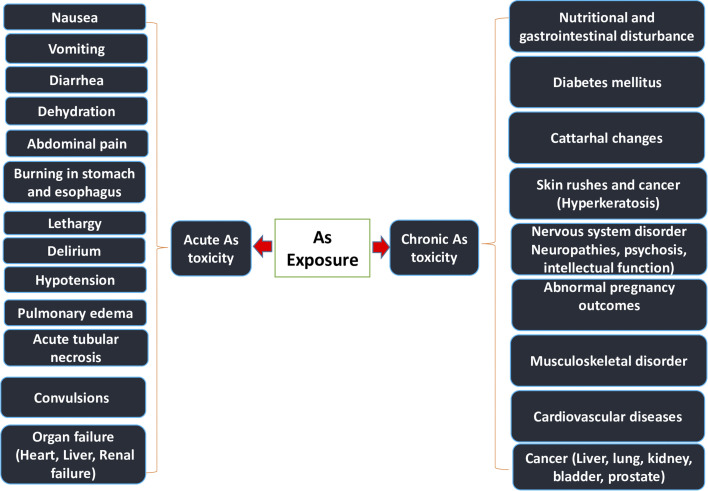
Consequence of As exposure on human organs.^37^

The WHO deemed this situation to be the “largest mass poisoning of a population in history” because Bangladesh as a whole experienced the worst As poisoning public health danger.^33^ In acute toxicity, As, a toxic metalloid, can cause nausea, vomiting, and severe diarrhea; in chronic toxicity, it can cause cardiovascular disease, diabetes, bladder cancer, and kidney cancer.^34^ Lung, bladder, kidney, skin, and liver malignancies, neurological disorders, cardiovascular diseases, hypertension, gangrene, diabetes, respiratory diseases, renal diseases, and reproductive diseases are among the prevalent adverse health effects of As exposure.^35^ The amount of ingested As, dietary status, length of exposure, and immune response of the individual are the key factors influencing the severity of As poisoning. Skin lesions, such as arsenicosis, are the telltale signs of chronic As exposure. Arsenicosis is a worldwide issue, not just a local one in Bangladesh, with Asian nations like Bangladesh, India, and China being the worst impacted.^36^ More effects of As exposure on the human body are illustrated in Fig. 3.

## 6 How As moves into plants and vegetables

In order to understand the mechanism supporting As transport, metabolism, and the detoxification mechanism responsible for scavenging As toxicity in diverse plants including rice, a number of genomes, proteomics, and metabolomic investigations have been conducted. Heavy metals and As can enter the body through specific routes. These routes include inhaling polluted dust, directly ingesting contaminated soil on the surface of foods, and ingesting plant-based foods that have been poisoned at the location of their growth. Vegetables have been reported to have the highest percentage of exposure to As, followed by fruit and fruit juices, rice, and other foods, in locations where there is no water contamination.^38^

As is easily accumulated by plants, which makes it easier for As to go from the soil into the food chain. In the oxic layer of soils, arsenate, which behaves chemically in a similar way to phosphate, is the main InAs species. As a result, As^V^ can enter plant species *via* the phosphate transporter system, whereupon the As^V^ in plant biomass may be changed into As^III^. The amount of phosphate in the soil affects the absorption and phytotoxicity of As. As can displace phosphate in soil particles at low concentrations, increasing absorption and phytotoxicity, whereas high concentrations of phosphate compete with As at root surfaces, decreasing uptake and phytotoxicity.^18,39^ In addition, As behaviour in the soil environment is influenced by microbial activities and hence As availability in the soil-plant system.^32^

## 7 As speciation in vegetables

### 7.1 Common extraction techniques and solvents

For information on accurate As speciation, it is necessary to maintain the concentration and chemical composition of the original species during the sample preparation and extraction procedures.^40^ The preferred extraction method for a given application reflects both the matrix and the target species. For the precise measurement of As species in vegetables and plants using HPLC-ICP-MS, a gentle and effective extraction procedure is needed.^18^ For the detection of As in vegetables, advanced techniques, such as shaking, sonication, accelerated solvent extraction and microwave-assisted extraction (MAE), have also been used (Table 2). However, for many matrices, MAE is an effective substitute for traditional methods because it provides acceptable and repeatable efficiency, shorter extraction duration, use of less solvent, and the ability to do numerous extractions quickly. Numerous investigations on the speciation of As have used this method. Due to the small number of variables involved, such as solvent selection, solvent volume, temperature, extraction time, power, and matrix characteristics, optimization is simple.^41,42^

**Table 2:** Summary of the most commonly used extraction procedures for extraction of As in plants and vegetables^a^

Matrix	Technique	Extraction solution	Extraction conditions	As species	Extraction efficiency (%)	Ref.
Welsh onion, spinach, romaine lettuce, leaf lettuce, leaf mustard, common sowthistle, pakchoi, sweet potato leaves, choy sum, pumpkin leaves	HPLC-ICP-MS	1% HNO_3_	Sonication, 25 °C	As^III^, As^V^, DMA	65.4–94.9	54
Garlic	HG-AFS	1 M H_2_SO_4_	Sonication for 20 min and EDTA, 20 min	As^III^, As^V^	97	55
Garlic	HG-AFS	1 M HClO_4_	Sonication for 20 min; and EDTA, 20 min	As^III^, As^V^	80	55
Garlic	HG-AFS	Methanol and water (1 : 1)	Sonication for 20 min; and EDTA, 20 min	As^III^, As^V^	96	55
*Pteris vittata*	HPLC-ICP-MS	2 mM NaH_2_PO_4_ and 0.2 mM	Sonication, 15 min	As^III^, As^V^	98 (fronds)	50
		Na_2_EDTA (pH 6)			<70 (roots)	
White mustard	HPLC-ICP-MS	Water	Sonication, 60 min	As^III^, As^V^, MMA, DMA	91 (roots)	45
					89 (stems)	
					50 (leaves)	
Leafy vegetables	HPLC-ICP-MS	1% HNO_3_	MAE, 90 °C, 1.5 h	As^III^, As^V^, DMA	77–105	52
Different vegetables	HPLC-ICP-MS	1% HNO_3_	MAE, 95 °C for 50 min	As^III^, As^V^, MMA, DMA	95–104	56
Spinach	IC-ICP-MS	Methanol and water (1 : 1)	MAE, 90 °C, 20 min	As^III^, As^V^, MMA, DMA	45	53
Spinach	IC-ICP-MS	PES	MAE, 90 °C for 20 min	As^III^, As^V^, MMA, DMA	100	53
Spinach	IC-ICP-MS	30 mM NH_4_H_2_PO_4_ (pH 5.6)	MAE, 90 °C for 20 min	As^III^, As^V^, MMA, DMA	101	53
Different compartments of broad bean	HPLC-ICP-MS	1% HNO_3_	MAE, 95 °C for 50 min	As^III^, As^V^, MMA, DMA	85–108	57
Carrots	LC-ICP-MS	Water	Accelerated solvent extraction, 100 °C	As^III^, As^V^, MMA, DMA	80–102	58
Pepper plant (fruit part)	HPLC-HG-ICP-MS	Water	Shaking for 14 h	As^III^, As^V^, MMA, DMA	87	59
Algae and aquatic plants	LC-ICP-MS	Water	Shaking for 16 h, 25 °C	As^III^, As^V^, MMA, DMA, glycerol-arsenosugars	5–127	60
Spinach	IC-ICP-MS	Water	Shaking for 1 h	As^III^, As^V^, MMA, DMA	80	53
Spinach	IC-ICP-MS	30 mM NH_4_H_2_PO_4_	Shaking for 1 h	As^III^, As^V^, MMA, DMA	70	53
Bush leaves	HPLC-HGAFS	Water–methanol	Shaking for 20 min, 60 °C	As^III^, As^V^, MMA, DMA	73	48

a HPLC: high performance liquid chromatography; ICP-MS: inductively coupled plasma mass spectrometry; HG-AFS: hydride generation atomic fluorescence spectrometry; IC: ion chromatography; LC: liquid chromatography.

Due to their ability to selectively hydrolyze the important parts of the cell, enzymes may be used as an extraction tool for As species. As a result, the amount of material needed could be greatly reduced, necessitating less sample dilution and enabling the analysis of As species that cannot be extracted using standard methods (water or water/methanol).^43^ Enzymes have also been employed to treat some food items; freeze-dried apple samples were treated with amylase. Amylase degrades the cellulose in freeze-dried apple samples, increasing the yields of As species during extraction. Acetonitrile–water extraction may then be used to complete the process.^43,44^ However, the extraction efficiency for As utilizing cellulase as the extraction agent was shown by cellulose to vary significantly for GBW10015 materials. For GBW10015-spinach, cellulase had an extraction efficiency of 119%. Additionally, some As species were retained in the residue because cellulase was unable to completely remove all the As from GBW10015-spinach. Because of this, cellulase is not a suitable extraction agent for extracting As species from plants and vegetables.^18^ There are many different extraction techniques that have been used for full, total inorganic, and total As speciation. The most commonly used extraction agents are water, methanol, methanol–water solvent systems, and occasionally, though rarely, acetonitrile–water solvent systems. Sequential extractions are also popular. Although water is an inexpensive and affordable solvent that can be used to remove As species in a plant and vegetable matrix, it is incapable of extracting InAs species (As^III^ and As^V^) that bind protein.^45^ Low recovery efficiency makes mixtures of methanol and water at various ratios ineffective for removing inorganic As from plant and vegetable tissues.^46–48^ Trifluoroacetic acid can increase the effectiveness of extraction, but it also has the potential to change the species of As.^49^ Although phosphate in the plasma could damage the cones of the mass spectrometer due to polymeric deposition and chlorine could interfere with the detection of ^75^As^+^ by forming the polyatomic species ^40^Ar^35^Cl^+^ in the subsequent ICP-MS analysis, diluted inorganic acid solutions (such as HNO_3_, H_3_PO_4_, and HCl) work well at extracting As species from plant tissues.^50,51^ It has been discovered that some mixed salt solutions, such as phosphate buffer solution (PBS) and protein extraction solution (PES), work effectively to remove various As species from plant samples.^52,53^Table 2 provides a summary of the main extraction media and extraction techniques utilized to remove As species from vegetables. It should be noted that several of these procedures have fairly low extraction efficiency and are typically time-consuming.

### 7.2 Techniques used for separation and measuring As species

#### 7.2.1 Methods used for separation of arsenicals

More than 300 As species have been identified using the most sophisticated analytical techniques.^61^ There is a need to separate these arsenical efficiently in real samples.

##### 7.2.1.1 Capillary electrophoresis (CE)

Due to its excellent separation efficiency and relatively gentle separation conditions, which would aid in conserving the original As species in samples, CE offers an alternative separation method to HPLC. Because CE is a separation method that operates at the nL min^−1^ level and because its (electro-osmosis dewatering) EOF is substantially lower than the uptake rate for traditional micro-concentric nebulizers of ICP-MS, coupling of CE and ICP-MS presents a difficult design challenge.^62^ Since CE has several distinct advantages over GC or HPLC methods, including high resolving power, quick and effective separation, minimal reagent consumption, and the potential for separation with only minor disruptions of the existing equilibrium between different species, CE is an appealing technique for elemental speciation.^63^ Separation of different organic and inorganic As species has been identified using CE.

##### 7.2.1.2 Chromatographic methods

###### 7.2.1.2.1 Ion chromatography

Cation-exchange chromatography is utilized with positively charged target molecules. Because the pH for chromatography is lower than the isoelectric point (pI), the molecule is positively charged. The stationary phase in this type of chromatography is negatively charged, and positively charged molecules are loaded to be dragged to it.^64^ Based on their physicochemical characteristics (p*K*_a_ values), As^V^, MMA, and DMA are deprotonated to create anionic species under neutral pH, whereas As^III^ exists as a neutral species. Due to variations in their anionic nature, anion-exchange chromatography is a potential alternative for separating these prevalent As species. Due to its low p*K*_a1_ (2.19) and p*K*_a2_ (6.98) values, As^V^ has the strongest negative charge in most mobile phases and elutes slowly. Given that As^III^ has high p*K*_a_ values (p*K*_a1_ = 9.23, p*K*_a2_ = 12.13, and p*K*_a3_ = 13.4), it often occurs as a neutral molecule, which results in relatively minimal retention. Many As compounds, including As^III^, As^V^, MMA, DMA, AsB, AsC, oxo-arsenosugars (oxoAsS), thio-arsenosugars (thioAsS), and phenylarsenicals, have been analysed using anion exchange chromatography. Strong anion-exchange columns, such as PRP-X100, are the most popular type of column used for As speciation studies.^65^

When the stationary phase is positively charged and negatively charged molecules are loaded to be attracted to it (*i.e.*, the pH for chromatography is larger than the pI), this process is known as anion-exchange chromatography. For the speciation investigation of positively charged As molecules, such AsB, AsC, TMAO, or TMA, cation-exchange chromatography is frequently utilized. Similar to anion exchange, cation-exchange chromatography operates by interacting with cationic analytes through the use of a negatively charged stationary phase. Stronger positively-charged analytes retain more information, which is directly correlated with the retention of arsenicals. In 15 minutes, Wolle *et al.* separated As^III^, MMA, As^V^, DMA, AsB, and TMAO using a strong cation-exchange (PRP-X200) column, with AsC and TMA co-eluting.^66^

###### 7.2.1.2.2 Ion-pair chromatography (IPC)

IPC has commonly been used for As speciation studies because it can distinguish between ionic and neutral species. Ion-pair reagents are used in the mobile phase of a typical reversed-phase column (C18) in the IPC process. While the hydrophobic area of the ion-pair reagent interacts with the stationary phase, the charged group of the reagent interacts with the analyte. Tetraalkylammonium, tetrabutylammonium, and tetraethylammonium are frequently utilized as ion-pair reagents for the speciation of anionic and neutral As species. The ion-pair separation of cationic and neutral As species frequently use alkyl sulfonates, such as hexane sulfonic acid and 1-pentane sulfonic acid. The two organic modifiers that are used most frequently are acetonitrile and methanol. Usually included in the mobile phase, they reduce retention duration and alter selectivity. The charged As species must pass through the ion-pair reagents and the hydrophobic stationary phase in order to interact with the conventional stationary phase, whereas the neutral As species can do so directly. In less than 12 minutes, Morita *et al.* developed a mixed ion-pair method using sodium butanesulfonate and tetramethylammonium hydroxide as the ion-pairing reagents to separate As^V^, As^III^, MMA, DMA, AsB, TMAO, TMA, and AsC.^67^ Eight As species, As^III^, As^V^, MMA, DMA, TMAO, tetramethylarsonium, AsC and AsB, have been identified in an extract of tree moss by ion-pair reversed phase HPLC.^68^

###### 7.2.1.2.3 Reversed-phase-liquid chromatography

Arsenolipids, which comprise fatty acids, phospholipids, phosphatidylcholines, fatty alcohols, and phosphatidylethanolamines, are particularly well analyzed by reversed-phase-liquid chromatography. Arsenolipids can be separated according to the number of carbons, the amount of double bonds, and other functional groups using standard C18 or C8 columns. Reversed-phase HPLC has been used with ICP-MS and ESI-MS to measure and identify several arsenolipids in fish and algae.^69,70^ As^III^, AsB, DMA, and an arsenosugar (oxo-arsenosugar-glycerol, As 328) in extracts of commercial kelp and bladderwrack have been separated and investigated using ion-pair-reversed-phase high-performance liquid chromatography (IP-RP-HPLC) coupled to particle beam-electron ionization mass spectrometry (PB-EIMS).^71^ The stability of three arsenolipids—As fatty acids AsFA-362 and AsFA-388, as well as As hydrocarbon AsHC-332—common components of algae—was examined in relation to sample storage and transport as well as their preparation for quantitative analyses.^70^

###### 7.2.1.2.4 Hydrophilic interaction liquid chromatography (HILIC)

Typically, anion-exchange columns and non-volatile solvents, for instance with phosphate buffers as mobile phase, are used to separate highly polar As compounds. Consequently, ESI-MS compatibility is only mild. So, a technique was created using hydrophilic interaction liquid chromatography (HILIC) for the separation of the main transformation products. Thus, compatibility of the used solvents for speciation analysis with both ICP-MS and ESI-MS was accomplished. In order to determine the quantity of the produced products and characterize the primary products developed, quantification of As-containing species was also carried out using high-resolution electrospray (EC)-HILIC-ICP-MS. Thirteen arsenicals were detected and separated using EC-HILIC-ICP-MS in research into roxarsone electrochemical transformation products.^72^ Xie *et al.* successfully *used* a zwitterionic HILIC column and ICP-MS/ESI-MS to separate nine organoarsenicals (*i.e.*, 3-nitro-4-hydroxyphenylarsonic acid (roxarsone, Rox), phenylarsonic acid (PAA), *p*-arsanilic acid (*p*-ASA), phenylarsine oxide (PAO), DMA, MMA, AsB, AsC and TMAO) within 45 min.^73^ Even though HILIC has significant potential for separating more As species in a single run, there are not many instances of it being used for As speciation.

###### 7.2.1.2.5 Size exclusion chromatography (SEC)

Size exclusion chromatography is not effective for speciation investigation of tiny As compounds because the size differences between many As species are tiny and they cannot be separated on an SEC column. The investigation of As interactions with big compounds, however, is particularly effective when using SEC. For instance, SEC is frequently used in an As–protein binding study to distinguish protein-bound from free As.^74,75^ With a focus on maintaining the intact proteins and their As bindings, a novel approach based on size exclusion chromatography linked to electrospray ionization mass spectrometry (SEC-ESI-MS) was developed in a study. Using SEC-ESI-MS, the simultaneous binding of phenylarsine oxide to five distinct peptides and proteins (glutathione, oxytocin, aprotinin, lactalbumin, and thioredoxin) were examined.^76^ SEC was also utilized to quantify As–biomolecule complexes in *Mus musculus* liver extracts.^77^ In order to separate and collect protein-bound As from free As, SEC was utilized; then, the protein-bound As was treated with hydrogen peroxide to liberate it.^78^ A combination of SEC coupled with ICP-MS, as well as three-dimensional excitation–emission matrix fluorescence spectroscopy combined with parallel factor analysis was used to investigate the roles of dissolved organic matter on As mobilisation and speciation in environmental water.^79^

### 7.3 Detection of As species

As can theoretically be found using any spectrometric detector that can identify an element specifically. Atomic absorption spectrometry (AAS), atomic fluorescence spectrometry (AFS), and mass spectrometry (MS) are the methods that are used most frequently.^80^

#### 7.3.1 Atomic absorption spectroscopy

An excitation source is necessary in atomic spectrometry in order to atomize or ionize the target analyte. These methods have the benefit of having innately sensitive and element-specific detection. In comparison to flame AAS, graphite furnace (GF)-AAS has been preferred for As research due to its 10–100-fold higher sensitivity. There have been reports of detection limits in the region of a few nanograms for both fraction collection and on-line coupling of HPLC with GFAAS.^11,81^ The baseline noise level was reduced by using a high-intensity boosted discharge hollow-cathode lamp, resulting in a lower LOD of 0.26 ppb for a sample volume of 16 L, or 4.2 pg of As.^82^

The AAS with vapour generation assembly (AAS-VGA) method is well known for As trace analysis. The conversion of As^V^ to As^III^ is necessary for appropriate analysis of the total As mixture (As^III^ + As^V^). The free As atoms that result from the conversion of As^III^ to AsH_3_ vapor and then free As are what provide the AAS absorption signal. This is accomplished using the AAS-attached vapor generation assembly, which contains a reduction channel filled with sodium borohydride and an acid channel filled with 10 M HCl.^83^

Due to its usefulness, simplicity, and affordability, AAS is an extensively used technique for metal measurement. However, sample pretreatment is frequently performed before the actual detection stage in order to enhance the metrological features of AAS, particularly the sensitivity and the detection limit.^84^ In reality, optical spectroscopy is a useful tool for identifying As^III^, As^V^, DMA, MMA, AsC, AsB and TAMO as well as for detecting considerable hydride arsenosugars and thioarsenate production when used in conjunction with various separation methods and chemical modifiers.^85,86^

#### 7.3.2 Atomic fluorescence spectroscopy

The use of HPLC in conjunction with atomic fluorescence spectrometry (AFS) for As speciation is now well established and effective. AFS is a good substitute for other atomic spectrometers that are frequently used in speciation research, like AAS and ICP-MS.^87^ Regarding performance factors like detection limits, reproducibility, repeatability, and sensitivity for As, AFS can compete with ICP-MS. Additionally, AFS provides reduced purchase and operating costs, quicker analysis warm-up times, and simple handling. Intriguing analytical properties offered by AFS include low LODs and a broad linear calibration range (from ppb to ppm).^88–90^

The drawbacks of the conventional HG borohydride/acid system have long been the subject of research into new vapor generation systems, such as electrochemical vapor generation (ECVG) and photochemical vapor generation (photo-CVG). The selective and quantitative conversion of As^III^ to AsH_3_ on the GSH-modified graphite electrode at an applied current of 0.4 A, while both As^III^ and As^V^ on the Cys-modified graphite electrode could generate AsH_3_ at an applied current of 0.6 A. This allowed for the speciation of As by coupling with AFS. By mixing 15 mg L^−1^ FeCl_3_ with acetic acid and formic acid, the ultraviolet vapor generation (UVG) of As has been increased around 10-fold.^91^ Additionally, under UV light, As^III^ and As^V^ could be transformed to volatile As species using a nano-Au/nano-TiO_2_ composite.^92^ AsH_3_ was produced at 0.6 A of applied current, but MMA and DMA could not form any or only a small amount of hydride under these conditions, achieving the speciation of As by coupling with AFS. HPLC-HG-AFS was employed to measure As^III^, As^V^, DMA and MMA in the roots, stems and fruits of strawberry plants.^93^

#### 7.3.3 Atomic emission spectroscopy (AES)

An alternative method for As speciation is atomic emission spectroscopy or optical emission spectroscopy (OES). Despite the ability of commercial ICP-OES to simultaneously determine many elements, the comparatively poor sensitivity can be a weakness in the study of As. On the other hand, OES is simple to miniaturize in order to create methods or devices for As analysis that can be used in the field.^94^ When very low limits of detection are not required, HPLC-ICP-AES is a reliable technology for As speciation. It has been concluded that detection limits better than 10 μg L^−1^ are needed for As^III^, DMA, and 20 g L^−1^ for As^V^. The method can also be combined with HG. However, it is important to keep in mind that not all As species can be identified in this way.^95^ HPLC-ICP-OES has been utilized to identify As species, including As^III^ and As^V^, in Chinese brake ferns with limits of detection of 0.032 mg L^−1^ As^III^ and 0.035 mg L^−1^ As^V^ per gram of dried fern material.^96^

#### 7.3.4 Inductively coupled plasma mass spectrometry (ICP-MS)

The majority of laboratories now use this technique for As speciation. Double focusing sector field ICP-MS allows for the immediate determination of desired elements without the need for pre-concentration or isolation. Double focusing ICP-MS offers great sensitivity over a broad linear range, low LODs, and the capability of multielement analysis.^97^ ICP-MS is the most widely used method for the detection of various As species due to its excellent sensitivity, selectivity, and wide dynamic range. Several strategies have been devised to minimize or diminish isobaric interference (identical mass isotopes of distinct elements present in the same sample) in the detection of As at a mass-to-charge ratio of 75.^98^ ICP-MS outperforms other techniques primarily due to its low detection limit range, 1–10 pg mL^−1^ for quadrupole instruments, broad linear dynamic range, speed, multi-element capabilities for many elements, and potential to apply isotopic studies (although not for As). ICP-MS for As speciation has certain limitations despite all these benefits. Without some type of previous separation, most frequently by HPLC, the use of ICP-MS does not provide direct molecular information and it is unable to identify specific As species.^99^

##### 7.3.4.1 Interference can be a problem in ICP-MS

When there is an isobaric overlap caused by polyatomic ions generated by the combination of two or more atoms, interference can be a problem in ICP-MS. The most abundant argon isotopes, ambient gases, and the solvents or acids used to prepare the sample all combine to generate the most significant polyatomic ions.^11^^40^As^35^Cl is a significant polyatomic interference species for As [As is monoisotopic *m*/*z* 75]. Refractory oxides may occur as a result of incomplete dissociation or recombination in colder plasma areas, particularly in the boundary layer surrounding the sampler cone.^100^ When polyatomic interference occurs, a collision/reaction cell can be used to reduce interfering ions by introducing a collision gas (such as helium), a reaction gas (such as oxygen, hydrogen, or CH_3_F), or a combination of two gases into the ICP-MS. ICP with triple quadrupole tandem mass spectrometry (ICP-QQQ) helps to eliminate isobaric interference, lessen background noise, and enhance selectivity compared to ordinary single quadrupole ICP-MS.^98^

There are numerous ways to reduce this interference issue in ICP-MS. Polyatomic interference can be reduced by introducing a different gas to the argon plasma, such as nitrogen, oxygen, air, helium, or hydrogen, which can also reduce the inherent polyatomic interference.^101^ Due to an increase in signal and a decrease in argon and O-based interference, adding nitrogen gas to an argon plasma has been proven to be quite successful.^102^ However, a more contemporary method that makes use of collision cell technology is currently offered on commercial instruments for the elimination of interference. In the case of As, a collision reaction cell with gases such H_2_, O_2_, NH_3_, CH_4_, NO, CO_2_, or C_2_H_4_ can reduce the ^40^Ar^35^Cl^+^ interference.^103–105^ Due to its sensitivity and capacity to resolve isobaric overlap, sector field (SF)-ICP-MS is possibly the most suitable option for elemental speciation research.^106^ As speciation in cucumber xylem sap is one instance of an As speciation study employing this method.^107^

#### 7.3.5 X-ray spectroscopic techniques

X-ray spectroscopic approaches are applied for As speciation analysis in As-rich biological samples with the least amount of sample preparation.^108^ Numerous X-ray spectroscopic techniques have been employed to measure total As and As speciation in various solid environmental and biological samples. This work looked into the feasibility of conducting speciation analysis on solid environmental samples without the need to extract the elemental species. As speciation has been investigated in plant material using X-ray absorption near-edge structure (XANES)^109,110^ and synchrotron radiation extended X-ray absorption fine structure (EXAFS).^111^

#### 7.3.6 Chemiluminescence (CL)

CL is the term for when a chemical reaction produces light. The CL created when As and ozone (O_3_) combine has been known for more than 30 years. In addition, the CL spectra of ozone and arsine gas are dissimilar. As is converted to arsine gas during CL analysis *via* hydride production, and the arsine gas is then sent to an ozone chamber *via* carrier gas flow for further reaction. With this method, As concentrations as low as 1 ppb can be detected. However, it is not particularly useful in the field because a carrier gas tank is required.^112^

CL detection is an optical detection method that provides great selectivity but at a price that is significantly less than that of atomic spectrometric methods. It has frequently been utilized in FI systems to identify both organic and inorganic species. Using luminol-based chemiluminescence detection or chemiluminescence produced by the redox reaction of As^III^ with permanganate in the presence of sodium hexametaphosphate, inorganic As species have both been successfully identified in FI systems. The relative detection limits for these techniques were 8 μg L^−1^, 100 μg L^−1^, and 0.3 μgL^−1^, respectively.^113^

## 8 As speciation in plants and vegetables

It has long been understood that As is a phytotoxic substance that perturbs the physiological and biochemical processes of plants. The toxicity depends on As speciation, typically, inorganic species are more poisonous to living things, such as plants, people, and other animals, than organic forms.^23^ Vegetables can absorb As from their surroundings (such as soil, irrigation water, and accumulated dust), leading to their future contamination.^114^ Therefore, for a population that consumes a lot of vegetables in As-contaminated areas, dietary intake may represent a significant As exposure pathway. As levels in a plant's edible compartments are influenced by the amount of As present in the soil, as well as the plant's capacity for accumulation and translocation.^115,116^ As is easily accumulated by vegetables, which makes it easier for As to go from the soil to the food chain. In the oxic layer of soils, As^V^, which behaves chemically in a similar way to phosphate, is the main InAs species. As a result, As^V^ can enter plant species *via* the phosphate transporter system, whereupon the As^V^ in the plant biomass may be changed into As^III^. The amount of phosphate in the soil affects the absorption and phytotoxicity of As. As can displace phosphate in soil particles at low concentrations, increasing absorption and phytotoxicity, whereas high concentrations of phosphate compete with As at root surfaces, decreasing uptake and phytotoxicity.^40^ Beside this, a protein transporter supports plants to absorb InAs. Usually, As compartments in roots then transfers to stem and grains.^117^Table 3 shows the literature on As species measured in vegetables and plants using different coupling instruments worldwide.

**Table 3:** Commonly utilized coupling techniques for detecting As species in various vegetables and plants in the literature^a^

Technique	Sample type	Contents of As species (μg kg^−1^)	Separation condition	Detection limit ng mL^−1^	Ref.
HPLC-ICP-MS	Welsh onion	As^III^: 140, As^V^: 200, DMA: 0	Hamilton, PRP-X100 anion exchange column; e mobile phase consisting of 8 mM (NH_4_)_2_HPO_4_ and 2 mM KNO_3_, pH 8	NG	54
	Spinach	As^III^: 2460, As^V^: 1930, DMA: 0			
	Romaine lettuce	As^III^: 8970, As^V^: 3590, DMA: 0			
	Leaf lettuce	As^III^: 6010, As^V^: 3130, DMA: 0			
	Leaf mustard	As^III^: 4700, As^V^: 5200, DMA: 0			
	Common sowthistle	As^III^: 340, As^V^: 470, DMA: 0.01			
	Pakchoi	As^III^: 4900, As^V^: 2400, DMA: 0			
	Sweet potato leaves	As^III^: 1210, As^V^: 340, DMA: 100			
	Choy sum	As^III^: 1370, As^V^: 650, DMA: 0			
	Pumpkin leaves	As^III^: 4490, As^V^: 3270, DMA: 330			
CE-ICP-MS	*Solanum lyratum* Thunb	As^III^: <LOD, As^V^: <LOD, DMA: <LOD	Running buffer solution 50 mmol L^−1^ H_3_BO_3_-12.5 mmol L^−1^ Na_2_B_4_O_7_, pH = 9.10; sheath liquid 5% (v/v) CH_3_OH	As^III^: 0.3, As^V^: 0.5, DMA: 0.6, AsB: 0.3, AsC: 0.2	62
		AsB: 420–1300, AsC: <LOD			
IC-ICP-MS	Spirulina powder samples	As^III^: 28–147, As^V^: 170–394	An anion exchange column (AEC, IonPac AS23), mobile phase 10.0 mmol L^−1^ (NH_4_)_2_HPO_4_ (pH 5.50); A cation-exchange column (CEC, IonPac CS12), mobile phase 0.10% v/v HCOOH (pH 3.00)	As^III^: 5, As^V^: 10, MMA: 5; DMA: 10	118
		DMA: 32–839; MMA: 67			
co-EOF-CZE	Kelp	As^III^, As^V^, DMA, MMA	The background solution was 100 mM borax buffer (pH 9.2), the separation voltage was −20 kV. The second stacking step used 60% MeOH	0.382 to 0.911	119
Ion pair HPLC-ICP-MS	Tree moss	As^III^: 48, As^V^: 53.7, DMA: 52.0, MMA: 53.7, TMAO: 27.0, tetra: 3.4, AsC: <LOD, AsB: 5.4, arsenosugar X: 42.9 (ng mL^−1^, as As)	CAPCELL-PAK C18 MG-II (250 mm × 4.6 mm, 5 μm), mobile phase A 10 mmol L^−1^ sodium butanesulfonate, 4 mmol L^−1^ TMAH and 4 mmol L^−1^ malonic acid, methanol/water (0.1/99.9, v/v), pH 3.0: mobile phase B 5 mmol L^−1^ ammonium acetate, methanol/water (1/99, v/v), pH 7.0: mobile phase C methanol/water (0.1/99.9, v/v), pH 3.0	As^III^: 0.06, As^V^: 0.06, DMA: 0.05, MMA, 0.06 TMAO: 0.07, Tetra: 0.07, AsC: 0.07, AsB: 0.07	68
IP-RP-HPLC-PB-EIMS	Kelp bladderwrack extracts	InAs: 6100, DMA: 960 (ng mL^−1^)	Dionex AS7 column, mobile phase 96 : 4H_2_O : MeOH w/0.1% trifluoroacetic acid, mobile phase (gradient) (A) 0.5 mmol L^−1^ HNO_3_, 2% MeOH (B) 50 mmol L^−1^ HNO_3_	As^III^: 0.03, DMA: 0.05, AsB: 0.008 and As 328 : 0.005	71
		InAs: 6200, DMA: 620 (ng mL^−1^)			
HPLC-ICP-OES	Chinese brake ferns	As^III^: NG, As^V^: NG	Hamilton PRP-X100 anion exchange column, mobile phase 30 mM NH_4_H_2_PO_4_ (pH = 6)	As^III^: 32, As^V^: 25	96
HPLC-ICP-MS	Leaf lettuce	As^III^: 31.8, As^V^: 93.1, DMA: 0.5	NG	NG	52
	Spring onion	As^III^: 95.3, As^V^: 142.3, DMA: 2.7			
	Celery	As^III^: 221.6, As^V^: 143.1, DMA: 1			
	Bok choy	As^III^: 23.4, As^V^: 87.9, DMA: <LOD			
	Napa cabbage	As^III^: 30.9, As^V^: 80.1, DMA: <LOD			
	Coriander	As^III^: 94.9, As^V^: 147.3, DMA: 1.0			
	Lettuce	As^III^: 79.4, As^V^: 114.3, DMA: 1.8			
	Purple-stem mustard	As^III^: 24.4, As^V^: 38.1, DMA: 0.5			
	Spinach	As^III^: 90.5, As^V^: 164.5, DMA: 6.1			
	Garlic chives	As^III^: 115.3, As^V^: 144.3, DMA: 2.0			
	Rapini	As^III^: 37.9, As^V^: 68.3, DMA: 0.3			
	Turnip green	As^III^: 131.4, As^V^: 175.6, DMA: <LOD			
	Choy sum	As^III^: 86.9, As^V^: 120.9, DMA: <LOD			
HPLC-ICP-MS	Carrots	As^III^: ND–230, As^V^: 16–96, MMA: ND, DMA: ND, AsB: ND	Column, waters IC-Pak anion HR; mobile phase, 10 mM ammonium carbonate, pH 10	As^III^: 8, As^V^: 6, MMA: 7, DMA: 12, AsB: 7 (ng g^−1^)	58
HPLC-HG-AFS	Carrot	As^III^: 90, As^V^: ND, DMA: ND, MMA: 22	Hamilton PRP-X100 column, mobile phase 10 mM K_2_HPO_4_/KH_2_PO_4_, pH 6.0 (isocratic)	As^III^: 17, As^V^: 14, DMA: 15, MMA: 11 (ng g^−1^)	120
	Kidney beans	As^III^: 42, As^V^: ND, DMA: ND, MMA: ND			
	Radish	As^III^: 94, As^V^: ND, DMA: ND, MMA: 60			
	Tomato	As^III^: 54, As^V^: ND, DMA: ND, MMA: ND			
	Onion	As^III^: 56, As^V^: ND, DMA: ND, MMA: ND			
	Betel nut	As^III^: 26, As^V^: ND, DMA: ND, MMA: ND			
	Cauliflower	As^III^: 60, As^V^: ND, DMA: ND, MMA: ND			
	Brinja	As^III^: 48, As^V^: ND, DMA: ND, MMA: ND			
	Potatoes	As^III^: 47, As^V^: ND, DMA: ND, MMA: 34			
HPLC-ICPMS	Potatoes	InAs: 8.44, MMA: <0.2, DMA: 0.648, AsB: <2, TMAO: <0.3, TMA: <0.1, AsC: <0.2 (values based on fresh weight)	Column: TSKgel super IC A/C, mobile phase: 0.35 mmol L^−1^ Na_2_SO_4_ (pH 3.1)	As^V^: 0.09, As^III^: 0.08, MMA: 0.04, DMA: 0.05, AsB: 0.01, TMAO: 0.07, TMA: 0.04, AsC: 0.05 (ng g^−1^)	121
	Pulses	InAs: 0.3, MMA: <0.1, DMA: <0.2, AsB: <2, TMAO: <0.2, TMA: <0.1, AsC: <0.1 (values based on fresh weight)			
	Vegetables	InAs: <0.1, MMA: <0.07, DMA: <0.008, AsB: <0.9, TMAO: <0.1, TMA: <0.06, AsC: <0.08 (values based on fresh weight)			
HPLC-ICP-MS	Broccoli	As^III^: 18.7–35.1, As^V^: 25.3–50.4, DMA: ND, MMA: ND	Hamilton PRP-X100 anion-exchange chromatographic column: mobile phase, (NH_4_)_2_HPO_4_ and water	As^III^: 0.068, As^V^: 0.073, DMA: 0.086, MMA: 0.065	122
	Caraway	As^III^: 153–200, As^V^: 132–207, DMA: ND–9.24, MMA: ND			
	Lettuce	As^III^: 84–136, As^V^: 92.8–142, DMA: ND–8.51, MMA: ND			
	Radish	As^III^: 32.8–53.3, As^V^: 58.1–60.9, DMA: ND–4.52, MMA: ND			
	Potato	As^III^: 19.1–23, As^V^: 23.9–26.3, DMA: 7.25–10.7, MMA: ND			
	Tomato	As^III^: 13.7–21.5, As^V^: 22.7–34.1, DMA: ND, MMA: ND			
	Zucchini	As^III^: 4.59–11.6, As^V^: 7.87–17.9, DMA: ND, MMA: ND			
	Green beans	As^III^: 17.9–26, As^V^: 28.7–36.6, DMA: ND, MMA: ND			
HG-AFS	Chards	As^III^: 89.2–90.6, As^V^: 14.2–15.3, DMA: 4.1–4.3, MMA: 3.5–3.7	Sonication at room temperature with H_3_PO_4_ 1 mol L^−1^ in the presence of 0.1% (w/v) Triton XT-114 and washing of the solid phase with 0.1% (w/v) EDTA	As^III^: 3.1, As^V^: 3.0, MMA: 1.9, DMA: 1.5 (ng g^−1^)	123
	Aubergine	As^III^: 20.6–20.9, As^V^: 61.0–61.9, DMA: 1.1–1.2, MMA: 1.2			
HPLC-ICP-MS	Broad bean-root	As^III^: 324, As^V^: 1585, DMA: 41, MMA: 68	Column, Hamilton resin PRP-X100, 10 μm particle size: mobile phase, 20 mM NH_4_H_2_PO_4_, pH 6.0	NG	18
	Broad bean-stem	As^III^: 35, As^V^: 62, DMA: 50, MMA: 44			
	Broad bean-leaf	As^III^: 91, As^V^: 232, DMA: <LOD, MMA: 101			
	Broad bean-pod	As^III^: 49, As^V^: 82, DMA: 70, MMA: 27			
	Broad bean-bean	As^III^: 9, As^V^: 24, DMA: 22, MMA: 55			
HG-AFS	Garlic	As^III^: 17.1–22.1, As^V^: 54.7–67.6	Ultrasound-assisted extraction procedure, 1 M H_2_SO_4_, 1 M HClO_4_, and methanol (99.98%) were used. A 0.1% (w/v) solution of the disodium salt of ethylenediaminotetraacetic acid	As^III^: 0.8, As^V^: 0.6 (ng g^−1^)	55
HPLC-ICP-MS	Herbal tea leaves-FF	As^III^: ND–293, As^V^: ND–554, DMA: ND, MMA: ND	Column, hamilton PRP-X100: mobile phase: mmol L^−1^ (NH_4_)_2_HPO_4_ (pH 6.0)	As^III^: 8.4,As^V^: 11.0, MMA: 8.6, DMA: 7.6 (ng g^−1^)	124
	Herbal tea leaves-S	As^III^: ND–461, As^V^: ND–625, DMA: ND, MMA: ND			
HPLC-ICP-MS	Lotus root	As^III^: <0.4^b^, As^V^: <0.6^b^, DMA: 3.5–7.9, MMA: <0.3^b^	Column, diamonsil (2) C18; mobile phase: A: 4 mM TBAH (pH 6.0)	As^III^: 0.078,As^V^: 0.15, MMA: 0.078, DMA: 0.081	125
			B: 4 mM TBAH + 20 mM Cys (pH 6.0)		

a NG: not given, co-electro-osmotic flow (co-EOF) capillary zone electrophoresis (CZE), FF: flower and fruits, S: strawberry.

b Values are less than limit of quantification; ETV: electrothermal vaporization.

## 9 Certified reference materials (CRM)

There has been an increased demand for various CRM types in chemical analysis during the past few years, as well as new CRM publications regarding their advances and certification. The increase in ISO/IEC 17025 accreditation serves as an example of the rising demand for traceable and trustworthy results in analytical chemistry. The usage of CRMs is highlighted among the several technological requirements in this quality system because of its application in many processes, including method validation, proficiency testing, uncertainty estimation, and quality control.^126^

Environmental control laboratories should utilize CRMs to test or verify the performance of their analytical methods in order to give accurate information and to comply with the data quality objectives of the legislation.^127^ As-containing CRMs have been produced, but the majority of them have total-element concentration certification. Because of the growing usage of species-specific measurement, species-specific CRM materials are now essential.^11^ Some certified materials used for As speciation in plants and vegetables are listed in Table 4.

**Table 4:** Some certified reference materials used to check validity of measuring total As and As species in plants and vegetables

Name of certified reference material	Extraction media	Certified value (μg kg^−1^)	Obtained value (μg kg^−1^)	Extraction efficiency (%)	Ref.
GBW10048 (GSB-26)	1% HNO_3_ under sonication at room temperature (25 °C)	390 ± 80	390 ± 30	100	54
GBW10015-spinach	1% HNO_3_, MAE	230 ± 30	249 ± 8	108	57
GBW 82301 (peach leaves)	Water–methanol, shaking for 20 min	340 ± 50	248 ± 10	73	48
BCR 279 sea lettuce (*Ulva lactuca*)	Water, shaking for 16 h	3090 ± 200	2900 ± 300	94	60
GBW10049-green onion	Water, MAE, 90 °C, 1.5 h	520 ± 110	489.9 ± 1.2	94	52
	80 mM NH_4_H_2_PO_4_ (pH = 7), shaking 90 °C, 1.5 h	520 ± 110	324.0 ± 2.3	62	52
	80 mM NH_4_H_2_PO_4_ (pH = 7), MAE, 90 °C, 1.5 h	520 ± 110	352.7 ± 9.2	68	52
	1% HNO_3_, shaking, 90 °C, 2 h	520 ± 110	419.3 ± 1.6	81	52
	1% HNO_3_, sonication, 2 h	520 ± 110	224.0 ± 2.8	43%	52
NCS-ZC-85006 (Tomato leaves)	MAE, HNO_3_ (69%)	1050 ± 130	940	89.5	128
CS-PR-2 (parsnip root powder)	MAE, HNO_3_ (69%)	30 ± 20	30	100	128
CRM NIST 1573a (Tomato leaves)	Ultrasound-assisted extraction procedure, 1 M H_2_SO_4_, 1 M HClO_4_, and methanol were used. A 0.1% (w/v) solution of the disodium salt of ethylenediaminotetraacetic acid	112 ± 4	110.2 ± 1.7	98.4	55

## 10 Conclusions

Millions of people worldwide rely heavily on edible plants and vegetables for food, yet when contaminated with toxins like As, they also pose the biggest threat to human health. The outcome of toxic As species present in vegetation on human health is of serious concern because several diseases are linked with toxic forms, especially if they surpass the allowable range. Although a certain limit for As in drinking water has been set, for vegetation this value has not yet been documented. Both can accumulate variable quantities of heavy metals as well as As species from environments such as water and soil, and anthropogenic processes. This review highlights an overview of available methods for measuring As species in edible plants and vegetables. Different chromatographic and non-chromatographic methods, and spectroscopic and coupled techniques were explained to identify and detect As species in edible plants and vegetables. The most promising and realistic methods of determining As species are recently coupled or hyphenated analytical techniques, including ICP-MS, LC-MS, and HPLC/ICP-MS.

## Conflicts of interest

There is no conflict of interest to declare.

## Supplementary Material
